# Integrated role of cardiac magnetic resonance and genetics in predicting left ventricular reverse remodelling in dilated and non‐dilated cardiomyopathy

**DOI:** 10.1002/ejhf.3671

**Published:** 2025-04-29

**Authors:** Martina Setti, Manuela Iseppi, Job A.J. Verdonschot, Jacopo G. Rizzi, Alessia Paldino, Carola Pio Loco Detto Gava, Giulia Barbati, Matteo Dal Ferro, Max F.G.H.M. Venner, Anne G. Raafs, Marta Gigli, Davide Stolfo, Antonio De Luca, Giulia De Angelis, Teresa M. Capovilla, Sharon Graw, Flavio L. Ribichini, Matthew Taylor, Luisa Mestroni, Stephane R.B. Heymans, Gianfranco Sinagra, Marco Merlo

**Affiliations:** ^1^ Center for Diagnosis and Treatment of Cardiomyopathies, Cardiothoracovascular Department Azienda Sanitaria Universitaria Giuliano‐Isontina (ASUGI), University of Trieste Trieste Italy; ^2^ European Reference Network for Rare, Low Prevalence and Complex Diseases of the Heart (ERN GUARD‐Heart); ^3^ Division of Cardiology Department of Medicine, University of Verona Verona Italy; ^4^ Division of Cardiology Ospedale Santa Chiara, Azienda Provinciale per i Servizi Sanitari della Provincia Autonoma di Trento Trento Italy; ^5^ Department of Cardiology, Cardiovascular Research Institute Maastricht (CARIM) Maastricht University Maastricht The Netherlands; ^6^ Department of Clinical Genetics Maastricht University Medical Center Maastricht The Netherlands; ^7^ Biostatistics Unit, University of Trieste Trieste Italy; ^8^ Department of Cardiology Catharina Hospital Eindhoven The Netherlands; ^9^ Division of Cardiology Department of Medicine, Karolinska Institutet Stockholm Sweden; ^10^ Cardiothoracic Department, Azienda Sanitaria Universitaria Friuli Centrale (ASUFC) University Hospital of Udine Udine Italy; ^11^ Cardiovascular Institute and Adult Medical Genetics Program University of Colorado Anschutz Medical Campus Aurora CO USA; ^12^ Center of Cardiovascular Research, Center for Molecular and Vascular Biology University of Leuven Leuven Belgium

**Keywords:** Dilated cardiomyopathy, Non dilated left ventricular cardiomyopathy, Left ventricular reverse remodelling, Arrhythmogenic genes, Ring‐like late gadolinium enhancement, Cardiac magnetic resonance

## Abstract

**Aims:**

Left ventricular reverse remodelling (LVRR) is a prognostic marker in patients with dilated (DCM) and non‐dilated left ventricular cardiomyopathy (NDLVC). The utility of combining late gadolinium enhancement (LGE) and genetic testing in predicting LVRR in DCM/NDLVC remains a knowledge gap. This study aimed to assess an integrated approach including LGE data and genetics to predict LVRR in DCM/NDLVC patients.

**Methods and results:**

This multicentre observational study included DCM/NDLVC patients with: (i) baseline echocardiographic left ventricular ejection fraction (LVEF) <50%; (ii) genetic testing; (iii) baseline cardiac magnetic resonance (CMR); (iv) 12‐month follow‐up echocardiographic data. LVRR was defined as LVEF increase ≥10% or LVEF ≥50% (if baseline LVEF <45%) at 12 months. Outcome measures were: (i) all‐cause mortality, heart transplant, or left ventricular assist device implantation (D/HT/LVAD); (ii) sudden cardiac death or major ventricular arrhythmias (SCD/MVA). Arrhythmogenic genes studied were *LMNA*, *DSP*, *FLNC,* and *RBM20*. Among 1757 DCM/NDLVC with genetic data, 616 met eligibility (462 DCM, 154 NDLVC; age 51 ± 14 years, 34% female). LVRR occurred in 314 patients (51%): 251 (54%) in DCM and 63 (41%) in NDLVC (*p* = 0.004). Independent predictors of LVRR within 1 year included titin truncating variants, absence of arrhythmogenic genes, and absence of LGE ring‐like pattern. In patients with LVEF <35%, only the presence of LGE ring‐like pattern and arrhythmogenic genes remained independently related to a lower rate of LVRR and increased SCD/MVA risk.

**Conclusion:**

In a large genetically and CMR characterized DCM/NDLVC cohort, arrhythmogenic genotypes and LGE ring‐like pattern were inversely related to LVRR, particularly in patients with LVEF <35%.

## Introduction

Left ventricular reverse remodelling (LVRR) is classically defined as the ability of the left ventricle to improve its systolic function, associated with dimensions and shape improvement when dilatation is present.^1^, ^2^, ^3^, ^4^ LVRR has been described in a consistent share of dilated cardiomyopathy (DCM) and non‐dilated left ventricular cardiomyopathy (NDLVC) patients presenting with left ventricular (LV) systolic dysfunction and treated with tailored therapies for heart failure with reduced ejection fraction (HFrEF).^5^, ^6^ Currently, myocardial tissue characterization is non‐invasively achievable through cardiac magnetic resonance (CMR) imaging, which detects late gadolinium enhancement (LGE) presence, pattern and quantification.^7^, ^8^ There is solid evidence showing an inverse relationship between LGE extent and LVRR.^9^, ^10^, ^11^ Additionally, an association between different clusters of pathogenetic variants and LVRR has been suggested in the DCM/NDLVC setting.^12^, ^13^ However, the integrated approach including genetics and CMR to predict LVRR in DCM and NDLVC remains unclear. To address this knowledge gap, we sought to evaluate a multiparametric approach which combines LGE with CMR, genetics and clinical parameters in predicting LVRR in a large cohort of patients with DCM and NDLVC presenting with systolic dysfunction.

## Methods

### Study population

We analysed all consecutive DCM and NDLVC patients presenting with LV ejection fraction (LVEF) <50%, enrolled in the multicentre Familial Cardiomyopathy Registry, including two centres (Cardiovascular Department, University of Trieste, Italy and Cardiovascular Institute, University of Colorado Anschutz Medical Campus, Aurora, CO, USA),^14^ and in the Maastricht Cardiomyopathy Registry.^15^ DCM and NDLVC were defined according to the 2023 European Society of Cardiology (ESC) guidelines for the management of cardiomyopathies.^16^ Coronary artery diseases, abnormal loading conditions, cardiotoxicity‐ and alcoholic‐related diseases were excluded.

All patients had echocardiographic evaluation at baseline and after 12‐month follow‐up, genetic testing, and CMR performed within 6 months of enrolment.

### Echocardiographic analysis

Baseline echocardiography was conducted at the time of initial diagnosis in the three referral centres or within 2 months for patients referred from other centres. The 12‐month echocardiographic evaluation was performed within a range of 9 and 15 months following enrolment.

LV dimensions and function were assessed in all patients according to the guidelines for cardiac chamber quantification.^17^ Apical four‐chamber and two‐chamber views were used for calculation of LV volumes and LVEF by Simpson's biplane method. In patients with atrial fibrillation, five consecutive beats were averaged. Reproducibility of echocardiographic LVEF calculation has been previously published.^18^, ^19^ Additionally, a reproducibility analysis was conducted on a random selection of 20 echocardiograms, which were independently analysed twice by two different cardiologists to assess interobserver variability. To evaluate the intraobserver variability, one cardiologist re‐analysed the echocardiograms at a separate time point.

### Gene variants and clusters

Next generation sequencing genetic analysis identified likely pathogenic/pathogenic (LP/P) gene variants, classified based on the American College of Medical Genetics and Genomics criteria (ACMG).^20^

Genotype‐negative patients, called ‘gene‐elusive’, were differentiated from genotype‐positive individuals. According to evidence from the literature, genotype‐positive patients were classified into specific genes or functional clusters, as previously reported.^2^, ^14^ Arrhythmogenic gene cluster included desmoplakin (*DSP*), lamin A/C (*LMNA*), filamin C (*FLNC*), and RNA binding motif protein 20 (*RBM20*).^16^

### Cardiac magnetic resonance and late gadolinium enhancement

All participants underwent CMR evaluation. CMR studies were performed on 1.5 or 3 T magnetic resonance scanners at each centre. Cine images in long‐axis (two‐, three‐ and four‐chamber) and short‐axis views were acquired using balanced steady‐state free precession pulse sequence. For the quantification of biventricular volumes, stroke volume, ejection fraction and LV mass, cine images were acquired in a stack of contiguous short‐axis slices from base to apex. Approximately 10 min after intravenous administration of 0.1–0.2 mmol/kg gadolinium‐based contrast agent, LGE images were acquired using segmented T1‐weighted inversion‐recovery prepared gradient‐echo or phase sensitive inversion recovery pulse sequences in the same views as those used for cine images, individually adjusting inversion time to optimize nulling of apparently normal myocardium.

LGE location was visually assessed and described as any septal (septal or combined septal/free‐wall), free‐wall, and ring‐like (defined as LV subepicardial/midmyocardial LGE involving at least three contiguous segments in the same short‐axis slice) patterns.^21^

### Study endpoint

The study endpoint was the achievement of LVRR, defined at the 12‐month echocardiographic follow‐up evaluation as a ≥10% absolute increase in LVEF or LVEF normalization (≥50%) in patients presenting with LVEF <45%.^2^, ^4^, ^13^

Moreover, two outcome secondary measures were considered: (i) all‐cause mortality, heart transplant, or LV assist device implantation (D/HT/LVAD); (ii) sudden cardiac death, or major ventricular arrhythmias (SCD/MVA). Patients who encountered outcomes prior to the 12‐month echocardiographic evaluation were excluded from the analyses. SCD was defined as witnessed immediate death with or without documented ventricular fibrillation or death occurring within 1 h of acute symptoms or nocturnal death with no antecedent history of immediate worsening symptoms. MVA were defined as resuscitated cardiac arrest, ventricular fibrillation, sustained ventricular tachycardia and appropriate implantable cardiac defibrillator (ICD) interventions.^22^

### Statistical analysis

Continuous variables were expressed as mean ± standard deviation or median (interquartile range) as appropriate. Categorical variables were expressed as percentages. Comparisons between groups were performed by the analysis of variance test on continuous variables or by the non‐parametric Mann–Whitney test, when necessary. Categorical variables were compared with the Chi‐square or Fisher's exact test, as appropriate. The cumulative probability of competing events of D/HTx/VAD and SCD/MVA over time was estimated compared between groups (LVRR+ vs. LVRR–), starting from the 12‐month echocardiographic evaluation for each patient. Univariable and multivariable odd ratios (OR) to identify predictors of LVRR were estimated using logistic regression analysis. Inter‐ and intraobserver variability was assessed by Bland–Altman plots. A *p*‐value <0.05 was considered statistically significant. All analyses were performed using R software (R Foundation for Statistical Computing, Vienna, Austria) and SPSS version 26.0 (SPSS Inc., Chicago, IL, USA).

## Results

### Study population

A total cohort of 1757 patients with available genetic testing was considered. Among them, 682 patients with CMR imaging were enrolled. The final study population consisted of 616 patients (95% probands) with available baseline and 12‐month echocardiograms (online supplementary *Figure* *S1*): 289 from the Trieste cohort, 303 from the Maastricht cohort, and 24 from the Denver cohort. At baseline, mean age was 51 ± 14 years, 209 (34%) were female and 333 (54%) were in New York Heart Association (NYHA) class II–IV. Most patients received optimal medical therapy following initial evaluation (*Table* *1*).

**Table 1:** Characteristics of the overall study population and according to genetic clusters

Variable	Overall cohort (*n* = 616)	Gene‐ elusive (*n* = 429, 70%)	Titin truncating variants (*n* = 102, 17%)	Arrhythmogenic genes (*n* = 39, 6%)	Cytoskeleton‐ Z disk genes (*n* = 11, 2%)	Sarcomeric genes (*n* = 28, 4%)	Other genes (*n* = 7, 1%)	*p*‐value
Clinical characteristics								
Age, years	51 ± 14	52 ± 13	48 ± 15	47 ± 13	47 ± 15	44 ± 16	45 ± 19	**<0.001**
Male sex, *n* (%)	407 (66)	278 (65)	73 (72)	23 (59)	9 (82)	20 (71)	4 (57)	0.5
NYHA class II–IV, *n* (%)	333 (54)	240 (56)	53 (52)	20 (51)	6 (55)	10 (36)	4 (57)	0.4
Type 2 diabetes mellitus, *n* (%)	41 (7)	32 (7)	3 (3)	2 (5)	2 (18)	2 (7)	0 (0)	0.3
CKD, *n* (%)	187 (30)	134 (31)	35 (34)	10 (26)	3 (27)	3 (11)	2 (29)	0.3
Family history of MVA, *n* (%)	84 (14)	41 (10)	18 (18)	13 (33)	2 (18)	8 (29)	2 (29)	**<0.001**
Probands, *n* (%)	586 (95)	418 (97)	92 (90)	35 (90)	10 (91)	25 (89)	6 (86)	**0.01**
Therapy								
Beta‐blockers								
Baseline, *n* (%)	516 (84)	358 (83)	90 (88)	32 (82)	10 (91)	22 (79)	4 (57)	0.4
12‐month follow‐up, *n* (%)	555 (90)	387 (90)	95 (93)	36 (92)	10 (91)	23 (82)	4 (57)	0.2
12‐month OMT, %	50 ± 30	49 ± 29	53 ± 32	48 ± 25	46 ± 37	51 ± 29	31 ± 9	0.8
ACEi								
Baseline, *n* (%)	429 (70)	295 (69)	75 (74)	29 (74)	8 (73)	18 (64)	4 (57)	0.8
12‐month follow‐up, *n* (%)	394 (64)	266 (62)	74 (73)	26 (67)	9 (82)	15 (54)	4 (57)	0.2
12‐month OMT, %	54 ± 32	57 ± 33	55 ± 32	39 ± 23	28 ± 9	55 ± 28	19 ± 9	**0.008**
ARB								
Baseline, *n* (%)	76 (12)	60 (14)	9 (9)	4 (10)	1 (9)	2 (7)	0 (0)	0.4
12‐month follow‐up, *n* (%)	77 (13)	61 (14)	8 (8)	4 (10)	0 (0)	4 (14)	0 (0)	0.1
12‐month OMT, %	51 ± 27	54 ± 27	39 ± 19	33 ± 19		63 ± 43		0.07
ARNi								
Baseline, *n* (%)	40 (6)	34 (8)	4 (4)	0 (0)	0 (0)	2 (7)	0 (0)	0.09
12‐month follow‐up, *n* (%)	79 (13)	64 (15)	9 (9)	4 (10)	0 (0)	2 (7)	0 (0)	0.1
12‐month OMT, %	68 ± 31	69 ± 30	63 ± 33	59 ± 39		69 ± 38		0.3
MRA								
Baseline, *n* (%)	285 (46)	213 (50)	45 (44)	12 (31)	2 (18)	11 (39)	2 (29)	**0.045**
12‐month follow‐up, *n* (%)	337 (55)	253 (59)	52 (51)	13 (33)	2 (18)	15 (54)	2 (29)	**0.002**
12‐month OMT, %	49 ± 29	50 ± 29	42 ± 25	45 ± 32	31 ± 15	46 ± 24	38 ± 18	0.2
SGLT2i, *n* (%)								
Baseline,	18 (3)	15 (3)	1 (1)	2 (5)	0 (0)	0 (0)	0 (0)	0.4
12‐month follow‐up	32 (5)	27 (6)	2 (2)	2 (5)	0 (0)	1 (4)	0 (0)	0.3
Loop diuretics, *n* (%)								
Baseline	247 (40)	178 (41)	48 (47)	10 (26)	1 (9)	8 (29)	2 (29)	**0.042**
12‐month follow‐up	178 (29)	125 (29)	36 (35)	9 (23)	1 (9)	5 (18)	2 (29)	0.2
Echo characteristics								
LVEF, %								
Baseline	33 ± 11	32 ± 10	32 ± 11	37 ± 11	34 ± 9	35 ± 11	40 ± 12	**0.04**
12‐month follow‐up	42 ± 10	42 ± 10	43 ± 10	40 ± 10	41 ± 10	43 ± 9	43 ± 10	0.6
LVEDVi, ml/m^2^								
Baseline	90 ± 27	93 ± 29	84 ± 28	85 ± 28	94 ± 29	74 ± 28	64 ± 13	**0.02**
12‐month follow‐up	75 ± 25	77 ± 26	71 ± 20	71 ± 21	84 ± 30	69 ± 18	68 ± 37	0.4
LAVi, ml/m^2^								
Baseline	46 ± 18	46 ± 18	49 ± 17	45 ± 17	54 ± 26	44 ± 13	31 ± 8	0.6
12‐month follow‐up	38 ± 14	39 ± 14	36 ± 11	43 ± 20	53 ± 20	33 ± 9	24 ± 10	**0.03**
LVRR, *n* (%)	314 (51)	218 (51)	64 (63)	9 (23)	4 (36)	16 (57)	3 (43)	**0.001**

ACEi, angiotensin‐converting enzyme inhibitor; ARB, angiotensin receptor blocker; ARNi, angiotensin receptor–neprilysin inhibitor; CKD, chronic kidney disease; LAVi, left atrial volume index; LVEDVi, left ventricular end‐diastolic volume index; LVEF, left ventricular ejection fraction; LVRR, left ventricular reverse remodelling; MRA, mineralocorticoid receptor antagonist; MVA, major ventricular arrhythmia; NYHA, New York Heart Association; OMT, optimal medical therapy; SGLT2i, sodium–glucose cotransporter 2 inhibitor.

Baseline characteristics differed between DCM (*n* = 462) and NDLVC (*n* = 154) patients, as shown in online supplementary *Table* *S1*. DCM patients were older (52 ± 13 vs. 47 ± 16 years, *p* = 0.0005), more symptomatic (NYHA class II–IV 60% vs. 38%, *p* < 0.0001), and had a lower incidence of family history of MVA (11% vs. 22%, *p* = 0.0007).

**Figure 1:**
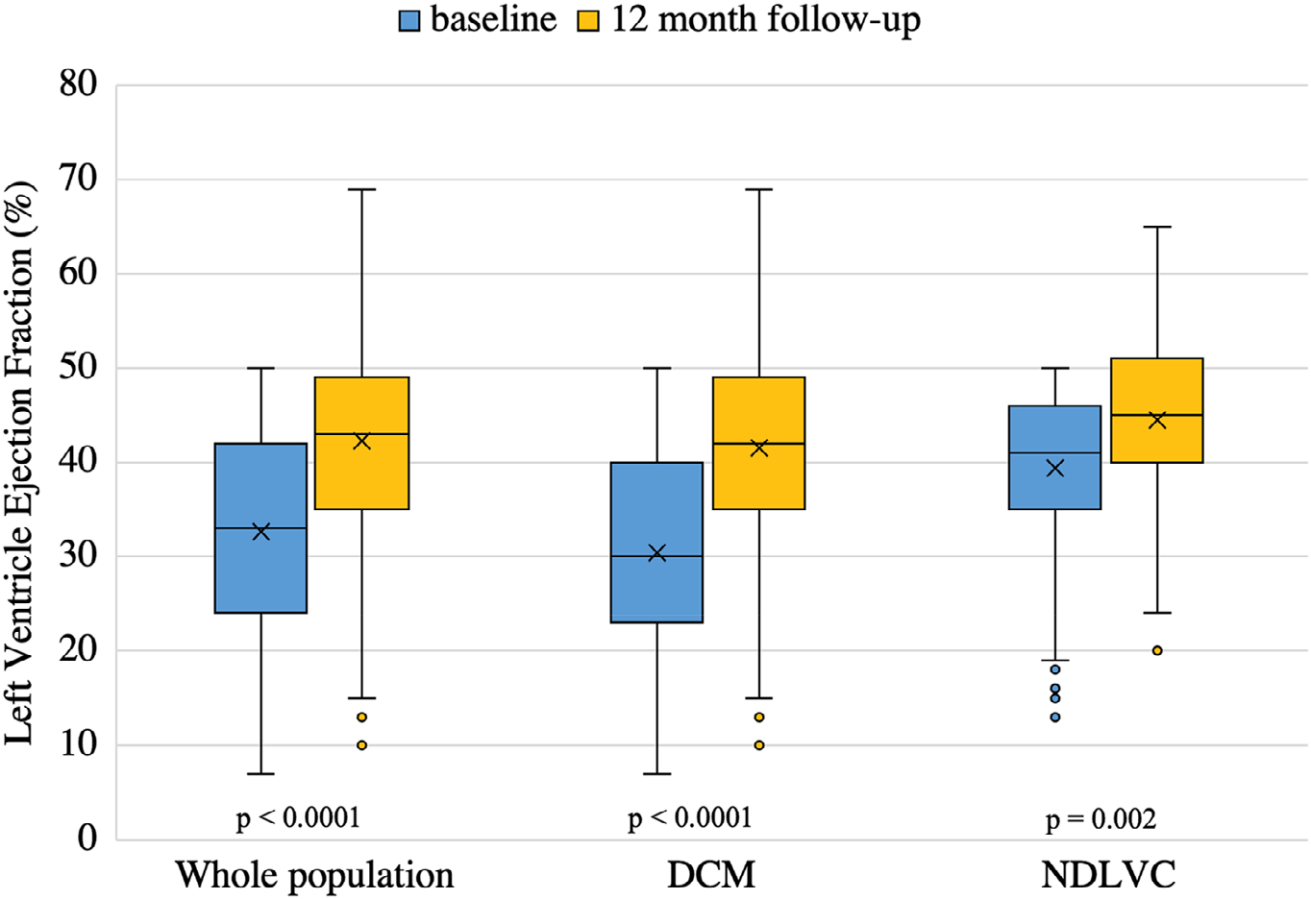
Left ventricular ejection fraction in the whole population, in patients with dilated (DCM) and non‐dilated left ventricular cardiomyopathy (NDLVC) at baseline and at 12‐month follow‐up.

Left ventricular reverse remodelling was observed in 314 (51%) patients: 251 out of 462 (54%) DCM and 63 out of 154 (41%) NDLVC patients (*p* = 0.004). Over the 12‐month follow‐up, mean LVEF significantly improved, rising from 33 ± 11% at baseline to 42 ± 10% (*p* < 0.0001) (*Figure* *1*). Specifically, DCM patients showed an increase in LVEF from 30 ± 10% to 42 ± 11% (*p* < 0.0001), whereas NDLVC patients improved from 39 ± 9% to 45 ± 9% (*p* = 0.002). Consistently, a significant reduction in LV end‐diastolic volume index (LVEDVi) was observed both in DCM and NDLVC (online supplementary *Table* *S1*). Left atrial volume index decreased from 46 ± 18 to 38 ± 14 ml/m^2^ (*p* < 0.0001), with a significant reduction in DCM (48 ± 18 to 38 ± 14 ml/m^2^, *p* = 0.0003), but not in NDLVC (40 ± 15 to 39 ± 16 ml/m^2^, *p* = 0.6). When considering LVRR across centres, it was observed in 46% of patients from Maastricht, 56% from Trieste, and 62% from Denver. No significant differences were found in medical therapy at 12 months or in the presence/pattern of LGE across the centres; however, the proportion of *TTNtv* carriers was higher in Trieste (*n* = 57, 20%) and Denver (*n* = 5, 21%) compared to Maastricht (*n* = 40, 13%).

Bland–Altman plots showed no significant differences between intraobserver measurements for either observer, nor between interobserver values (for interobserver variability: mean difference −0.75 [−1.16 to 2.76], agreement 0.86, *p* = 0.4; for intraobserver variability: mean difference −0.25 [−1.24 to −0.74], agreement 0.97, *p* = 0.4).

### Genetic clusters and left ventricular reverse remodelling

Genetic testing identified LP/P genetic variants in 187 patients (30%). The LP/P variant genes included in each functional genetic clusters are listed in online supplementary *Table* *S2*. The most frequently involved genes were *TTNtv*, found in 102 genotype‐positive patients (17% of the whole cohort), followed by motor sarcomeric genes (28 patients, 4%), *FLNC* (16 patients, 3%), cytoskeleton‐Z disk genes (11 patients, 2%), *LMNA* (11 patients, 2%), *DSP* (8 patients, 1%), and *RBM20* (4 patients, <1%) (online supplementary *Figure* *S2*). Clinical and echocardiographic characteristics of the cohort according to genetic clusters are detailed in *Table* *1*.

**Figure 2:**
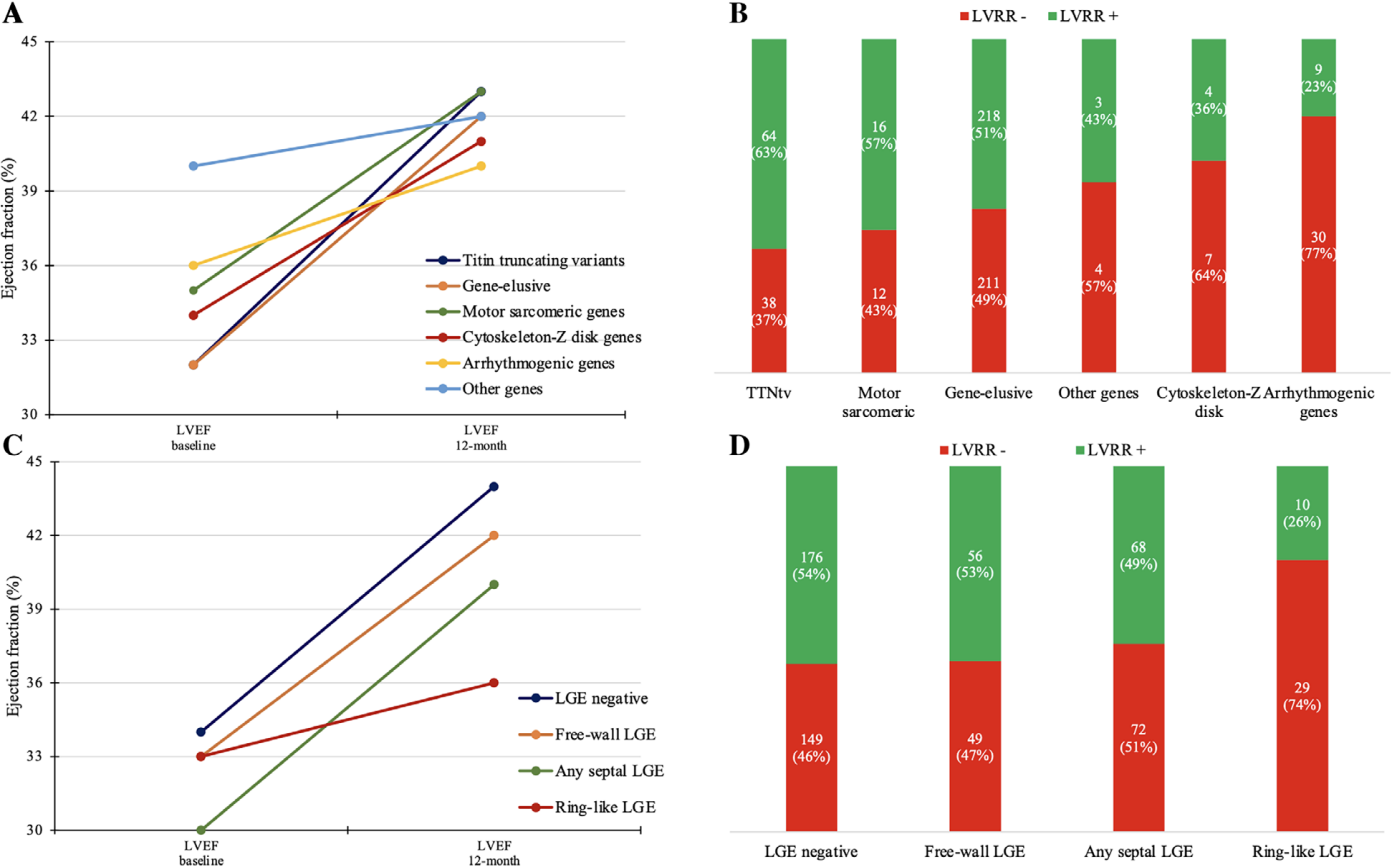
Trends of left ventricular ejection fraction (LVEF) from baseline to 12‐month follow‐up and rates of left ventricular reverse remodelling (LVRR), according to genetic clusters (*A* and *B*) and late gadolinium enhancement (LGE) location (*C* and *D*). TTNtv, titin truncating variant.

Left ventricular reverse remodelling occurred more frequently in *TTNtv* carriers (63%), followed by sarcomeric genes (57%), gene‐elusive (51%), cytoskeleton‐Z disk genes (36%) and, lastly, arrhythmogenic gene carriers (23%, including 3 [27%] *LMNA*, 2 [25%] *DSP*, 3 [19%] *FLNC*, 1 [25%] *RBM20*), *p* = 0.001 for all comparisons). Of note, *TTNtv* carriers showed the lowest LVEF (32 ± 11%) at baseline and the highest LVEF (43 ± 10%) at follow‐up compared to other subgroups (*Figure* *2A,B*). Genetic cluster distribution did not differ significantly between DCM and NDLVC (*p* = 0.1) (online supplementary *Table* *S1*).

### Late gadolinium enhancement location and left ventricular reverse remodelling

Late gadolinium enhancement at CMR was detected in 291 (47%) patients. Free‐wall LGE was observed in 17%, any septal LGE (only septal or combined septal/free‐wall LGE) in 23% and ring‐like LGE pattern in 6%. The location of LGE was unspecified in 7 patients. Clinical and echocardiographic characteristics of the patients according to LGE location are detailed in *Table* *2*. LVRR was found only in 26% of LGE ring‐like pattern, 49% of any septal LGE, 53% of free‐wall LGE, and 54% of no‐LGE patients (*p* = 0.007 for all comparisons) (*Figure* *2C,D*). Of note, in the LGE ring‐like subgroup, a milder LV dilatation at baseline by echocardiography (LVEDVi 80 ± 29 ml/m^2^) was present. LGE location was similar between DCM and NDLVC (*p* = 0.4) (online supplementary *Table* *S1*).

**Table 2:** Characteristics of the study population according to late gadolinium enhancement location

Variable	No LGE (*n* = 325, 53%)	Any septal LGE (*n* = 140, 23%)	Free‐wall LGE (*n* = 105, 17%)	Ring‐like (*n* = 39, 6%)	*p*‐value
Clinical characteristics					
Age, years	49 ± 14	53 ± 12	53 ± 13	49 ± 13	**0.02**
Male sex, *n* (%)	204 (63)	99 (71)	77 (73)	23 (60)	0.09
NYHA class II–IV, *n* (%)	162 (50)	81 (58)	64 (61)	22 (56)	0.05
Type 2 diabetes mellitus, *n* (%)	18 (6)	14 (10)	4 (4)	4 (10)	0.2
CKD, *n* (%)	88 (27)	42 (30)	42 (40)	15 (38)	0.2
Family history of MVA, *n* (%)	45 (14)	20 (14)	14 (13)	4 (10)	0.9
Probands, *n* (%)	308 (95)	131 (94)	101 (96)	39 (100)	0.2
Therapy					
Beta‐blockers					
Baseline, *n* (%)	262 (81)	123 (88)	91 (87)	35 (90)	0.1
12‐month follow‐up, *n* (%)	280 (86)	134 (96)	99 (94)	36 (92)	**0.003**
12‐month OMT, %	48 ± 29	54 ± 30	49 ± 30	51 ± 31	0.3
ACEi					
Baseline, *n* (%)	220 (68)	102 (73)	71 (68)	30 (77)	0.5
12‐month follow‐up, *n* (%)	209 (64)	87 (62)	65 (62)	27 (69)	0.8
12‐month OMT, %	55 ± 33	56 ± 31	56 ± 33	46 ± 30	0.5
ARB					
Baseline, *n* (%)	40 (12)	19 (14)	12 (11)	4 (10)	0.9
12‐month follow‐up, *n* (%)	47 (14)	15 (11)	10 (10)	4 (10)	0.5
12‐month OMT, %	50 ± 27	54 ± 28	47 ± 27	60 ± 34	0.7
ARNi					
Baseline, *n* (%)	13 (4)	14 (10)	11 (10)	2 (5)	**0.03**
12‐month follow‐up, *n* (%)	26 (8)	28 (20)	20 (19)	5 (13)	**0.001**
12‐month OMT, %	66 ± 32	69 ± 32	68 ± 29	70 ± 35	0.9
MRA					
Baseline, *n* (%)	136 (42)	67 (48)	53 (50)	24 (62)	0.07
12‐month follow‐up, *n* (%)	168 (52)	82 (59)	58 (55)	24 (62)	0.426
12‐month OMT, %	49 ± 28	49 ± 32	43 ± 25	49 ± 30	0.4
SGLT2i, *n* (%)					
Baseline	6 (2)	5 (4)	5 (5)	2 (5)	0.4
12‐month follow‐up	11 (3)	12 (9)	7 (7)	2 (5)	0.1
Loop diuretics, *n* (%)					
Baseline	106 (33)	66 (47)	52 (50)	19 (49)	**0.001**
12‐month follow‐up	68 (21)	51 (36)	39 (37)	16 (41)	**<0.001**
Echo characteristics					
LVEF, %					
Baseline	34 ± 10	30 ± 10	32 ± 11	33 ± 11	**0.02**
12‐month follow‐up	44 ± 10	40 ± 10	42 ± 10	36 ± 11	**<0.001**
LVEDVi, ml/m^2^					
Baseline	90 ± 27	94 ± 26	89 ± 30	80 ± 29	0.2
12‐month follow‐up	73 ± 22	81 ± 27	74 ± 27	77 ± 29	0.08
LAVi, ml/m^2^					
Baseline	43 ± 16	51 ± 17	46 ± 16	53 ± 27	**0.004**
12‐month follow‐up	36 ± 12	41 ± 15	39 ± 16	46 ± 20	**0.002**
LVRR, *n* (%)	176 (54)	68 (49)	56 (53)	10 (26)	**0.007**

ACEi, angiotensin‐converting enzyme inhibitor; ARB, angiotensin receptor blocker; ARNi, angiotensin receptor–neprilysin inhibitor; CKD, chronic kidney disease; LAVi, left atrial volume index; LGE, late gadolinium enhancement; LVEDVi, left ventricular end‐diastolic volume index; LVEF, left ventricular ejection fraction; LVRR, left ventricular reverse remodelling; MRA, mineralocorticoid receptor antagonist; MVA, major ventricular arrhythmia; NYHA, New York Heart Association; OMT, optimal medical therapy; SGLT2i, sodium–glucose cotransporter 2 inhibitor.

### Predictors of left ventricular reverse remodelling

In multivariable analysis (*Table* *3*, see online supplementary *Table* *S3* for univariable analysis), the presence of *TTNtv* was associated with LVRR (odds ratio [OR] vs. other genes: 1.57 [95% confidence interval 1.01–2.45], *p* = 0.04). Conversely, carrying arrhythmogenic genes (OR vs. other genes: 0.27 [0.12–0.60], *p* = 0.001) or LGE ring‐like pattern (OR 0.33 [0.16–0.71], *p* = 0.004) were negatively associated with LVRR (*Graphical Abstract*). This finding remained consistent when a sensitivity analysis excluding the 24 patients from the Denver centre was performed, confirming no significant impact from their exclusion. Remarkably, in the sensitivity multivariable analysis restricted to patients with baseline LVEF <35%, only LGE ring‐like pattern (OR 0.24 [0.09–0.65], *p* = 0.005) and arrhythmogenic genes (OR vs. other genes: 0.24 [0.07–0.83], *p* = 0.02) emerged as negative predictors. Conversely, in patients with LVEF ≥35%, the presence of *TTNtv* was the only independent predictor of LVRR (OR vs. other genes: 2.15 [1.09–4.25], *p* = 0.03) (online supplementary *Table* *S4*).

**Table 3:** Predictors of left ventricular reverse remodelling in univariable and multivariable analysis

	Univariable analysis			Multivariable analysis		
	OR	95% CI	*p*‐value	OR	95% CI	*p*‐value
Age	1.01	0.99–1.02	0.2			
Male sex	1.36	0.97–1.90	0.07			
NYHA class II–IV	1.06	0.76–1.48	0.7			
Type 2 diabetes mellitus	1.41	0.74–2.69	0.3			
CKD	1.31	0.91–1.86	0.1			
Beta‐blockers ‐ 12‐month follow‐up	1.01	0.59–1.71	0.9			
ACEi/ARB/ARNi ‐ 12‐month follow‐up	1.49	0.90–2.45	0.1			
LBBB	1.45	0.97–2.17	0.2			
Systolic blood pressure	1.01	0.99–1.01	0.3			
TTNtv^a^	1.78	1.15–2.75	**0.009**	1.57	1.01–2.45	**0.04**
Arrhythmogenic genes^a^	0.27	0.13–0.57	**0.0007**	0.27	0.12–0.60	**0.001**
LGE ring‐like pattern	0.31	0.15–0.69	**0.002**	0.33	0.16–0.71	**0.004**

ACEi, angiotensin‐converting enzyme inhibitor; ARB, angiotensin receptor blocker; ARNi, angiotensin receptor–neprilysin inhibitor; CI, confidence interval; CKD, chronic kidney disease; LBBB, left bundle branch block; LGE, late gadolinium enhancement; NYHA, New York Heart Association; OR, odds ratio; TTNtv, titin truncating variant.

^a^ OR versus other genes.

In DCM, *TTNtv*, LGE ring‐like pattern, and arrhythmogenic genes were confirmed predictors in univariate analysis, whereas only LGE ring‐like pattern was inversely associated with LVRR in NDLVC (OR 4.63 [1.01–21.48], *p* = 0.05).

### Interplay between genetic clusters, late gadolinium enhancement distribution and left ventricular reverse remodelling

**Figure 3:**
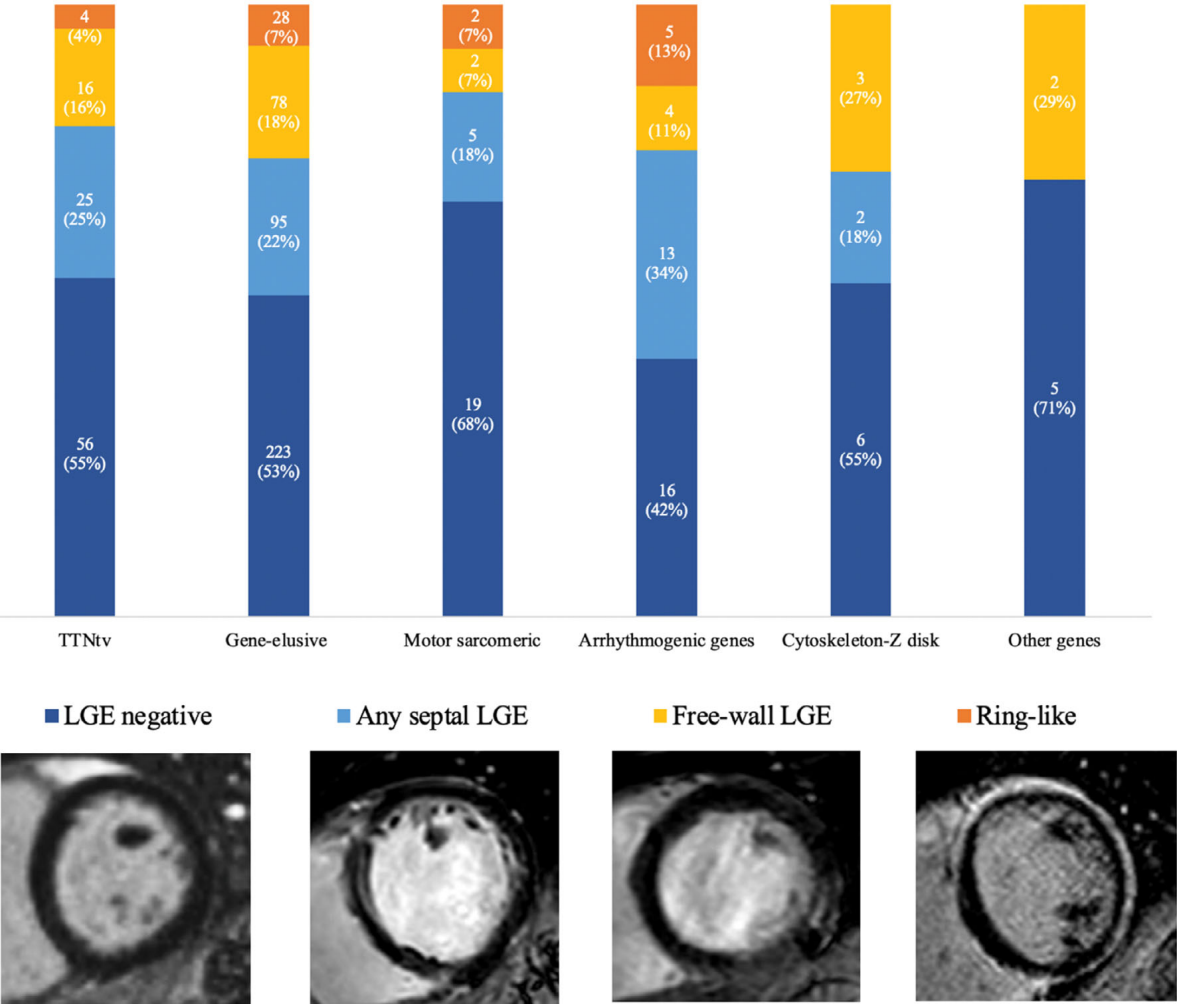
Late gadolinium enhancement (LGE) location in different functional gene clusters. TTNtv, titin truncating variant.

*Figure* *3* shows the distribution of genetic clusters across different LGE locations. Notably, only 4% of *TTNtv* carriers presented a LGE ring‐like pattern. Conversely, 13% of patients carrying arrhythmogenic gene variants presented LGE ring‐like pattern. Interestingly, 28 out of 39 patients (72%) with LGE ring‐like pattern were gene‐elusive, consistent across the Trieste (18 out of 25, 72%) and Maastricht (10 out of 14, 71%) cohorts.

At an exploratory analysis, *TTNtv* carriers showed increasing LVRR rates from those with LGE ring‐like pattern (50%) to those with free‐wall LGE (*n* = 11, 69%). Among arrhythmogenic gene carriers, LVRR rates were much lower among subgroups (from 0% in those with LGE ring‐like pattern to 50% in those with free‐wall LGE) (online supplementary *Figure* *S3*).

### Prognostic secondary endpoints

Over a median follow‐up of 7.2 years (interquartile range 3.5–11.0), 77 (13%) patients met the survival endpoint (i.e. 68 deaths, 8 HTx, 1 LVAD) and 74 (12%) the arrhythmic endpoint (i.e. 16 SCD, 58 MVA). Eight patients (1%) were excluded from the analysis as they experienced MVA before the 12‐month follow‐up echocardiographic evaluation. Among them, six had DCM and two had NDLVC. Regarding LGE patterns, 3 (38%) had a ring‐like pattern, 2 (25%) had no LGE, 2 (25%) had any septal LGE, and 1 (12%) had free‐wall LGE. Genetically, half of the excluded patients (*n* = 4) were gene‐elusive, two carried arrhythmogenic genes (*LMNA*, *DSP*) and none carried *TTNtv*.

DCM/NDLVC patients with LVRR at 12‐months follow‐up had a significantly better long‐term prognosis (*p* < 0.001) and a lower arrhythmic risk (*p* = 0.04) (online supplementary *Figure* *S4*). Particularly, an elevated arrhythmic risk was confirmed in patients carrying arrhythmogenic gene variants or presenting LGE ring‐like pattern (hazard ratio [HR] 2.71 [1.39–5.31], *p* = 0.004 for arrhythmogenic genotype; HR 3.52 [1.79–6.93], *p* = 0.0003 for LGE ring‐like pattern), whereas *TTNtv* carriers did not show a significant elevation in risk (HR 1.35 [0.78–2.33], *p* = 0.3) (*Graphical Abstract*). The associations with arrhythmic risk were confirmed analysing only patients with baseline LVEF <35% (HR 4.14 [1.62–10.59], *p* = 0.003 for arrhythmogenic genotype; HR 4.57 [1.91–10.93], *p* = 0.0006 for LGE ring‐like pattern).

In the sub‐analysis comparing DCM with NDLVC, the survival outcome was similarly favourable in both groups for patients with LVRR (*p* = 0.002 vs. *p* = 0.05) (online supplementary *Figure* *S5A*, *B*). However, the reduction in the risk of SCD/MVA was observed only in DCM patients with LVRR (*p* = 0.02 vs. *p* = 0.7) (online supplementary *Figure* *S5C*, *D*).

## Discussion

The current study investigated the role of both LGE at CMR and genetic background in predicting LVRR in a large, well‐characterized DCM and NDLVC population. The key novel findings are the following. First, more than half patients (51%) showed LVRR at 12‐month follow‐up, with a significantly higher probability in DCM compared to NDLVC patients. Second, *TTNtv* carriers exhibited a higher likelihood of LVRR, mostly in patients with LVEF 35% to 50% at baseline. On the other side, arrhythmogenic genes (i.e. *LMNA*, *DSP*, *FLNC*, and *RBM20*) and LGE ring‐like pattern emerged as high‐risk hallmarks for failed LVRR and, subsequently, MVA, especially in patients starting with LVEF <35%. Those findings were generally consistent in DCM and NDLVC patients. Finally, LGE location varied across different genotypes (*Figure* *3*). These findings reinforce the need to deeply and early characterize DCM and NDLVC patients, as recommended by the recent ESC guidelines for the management of cardiomyopathies.^16^

### Prevalence of left ventricular reverse remodelling

The high proportion (51%) of DCM/NDLVC patients with LVRR at 12‐month follow‐up, compared to previous reports (ranging from 35% to 52%),^4^, ^23^, ^24^ likely reflects the impact of recently introduced pharmacological and non‐pharmacological treatments in HFrEF patients (namely, angiotensin receptor–neprilysin inhibitors and sodium–glucose cotransporter 2 inhibitors). This finding underscores the highly dynamic nature mostly of DCM but also of NDLVC and the potential for positive remodelling through therapeutic interventions.^25^ Significant clinical consequences are possible, from the lower need for implantable devices compared to the recent past, to the need for more personalized arrhythmic risk stratification in the next future.

In our cohort, NDLVC with reduced LVEF appeared as an early stage of DCM, affecting younger, and less symptomatic patients. The higher baseline LVEF in NDLVC patients translates into a lower improvement in LV systolic function, highlighting the influence of initial LVEF on recovery outcomes^23^ and the need for tailored definitions of LVRR specifically referred to DCM and NDLVC. Further studies on larger cohorts are needed to better understand the entire clinical spectrum of NDLVC and the possible crossover between NDLVC and DCM during long‐term follow‐up.

### Genetic clusters and left ventricular reverse remodelling

We found that 63% of *TTNtv* carriers experienced LVRR, corroborating the evidence that the majority of *TTNtv*‐related DCM/NDLVC recover LVEF under treatment after 12 months.^13^, ^26^ As additional environmental factors (i.e. peripartum, alcohol, chemotherapy) can contribute through a ‘second‐hit’ role unmasking the dilated phenotype in *TTNtv* carriers,^27^, ^28^, ^29^ and removing modifier environmental contributions with pharmacological and non‐pharmacological treatments is likely to have a positive impact on systolic dysfunction recovery. Notably, carriers of arrhythmogenic genes encountered lower likelihood of LVRR (23%), confirming the high arrhythmic risk of this subgroup.^16^ These findings further emphasize the utility of genetic data in tailoring therapeutic strategies for better prognostic stratifications of DCM and NDLVC patients.

### Late gadolinium enhancement location and left ventricular reverse remodelling

While specific LGE locations have been linked to an increased risk of SCD,^30^ less is known about the association between LGE location and LVRR. Previous studies have reported the absence or lower extent of LGE as independently associated with LVRR.^6^, ^31^, ^32^ Interestingly, 149 (46%) DCM/NDLVC patients, despite not presenting LGE, also did not exhibit LVRR at 12 months, indicating that the absence of LGE alone does not guarantee the complex reverse remodelling process. When considering the specific LGE location in the whole cohort, a significant difference between groups was observed. Specifically, 53% of patients with free‐wall LGE experienced LVRR, in contrast with only one‐quarter of patients with LGE ring‐like pattern (*Figure* *3*). This result recognized that not only the extent of LGE but also its location, and ring‐like pattern may be related with the reverse remodelling process, both in DCM and NDLVC. Future focused and larger studies are needed to confirm this hypothesis.

### Interplay between genetic clusters, late gadolinium enhancement distribution and left ventricular reverse remodelling

Specific genetic backgrounds were differently associated with LGE locations. Only 4% of *TTNtv* carriers had a LGE ring‐like pattern compared to 13% of arrhythmogenic gene carriers, and *TTNtv* carriers with a ring‐like LGE pattern showed a lower likelihood of LVRR (50%) compared to other LGE distributions. However, consistently lower rates of LVRR were found in arrhythmogenic genes and none of the patients with both arrhythmogenic gene variants and ring‐like LGE pattern achieved LVRR. Interestingly, in our cohort, 72% of DCM/NDLVC patients displaying a LGE ring‐like pattern were gene‐elusive. This finding may seem inconsistent with previous studies showing frequent LP/P variants in genes such as *DSP, FLNC*, and other arrhythmogenic genes^33^, ^34^, ^35^ in patients presenting LGE ring‐like pattern. However, previous cohorts focusing on genotype in LGE ring‐like pattern excluded genotype‐negative patients^33^, ^34^, ^35^, ^36^, enrolled only a small proportion of patients undergoing genetic testing^32^ (resulting in a younger patient population), or applied a more stringent definition of LGE ring‐like pattern.^36^ The absence of LP/P genetic variants in our LGE ring‐like cohort suggests that these patients might harbour other underlying genetic factors, such as polygenic contributions, variants of uncertain significance, and non‐coding regions. Additionally, we cannot exclude that a portion of these patients may fall within a post‐inflammatory aetiology.^37^ The likelihood of obtaining negative results in patients with LGE ring‐like pattern was confirmed in both the Trieste and Maastricht cohorts. A well‐defined classification of the LGE ring‐like pattern will enable more comparable and targeted studies to improve our understanding of this phenomenon.

Finally, different LGE locations across different genotypes add complexity to the understanding of phenotypic expressions in DCM, suggesting a genotype‐specific impact on myocardial fibrosis patterns,^33^ which are strictly linked to disease progression and treatment response.

### Clinical implications

Although confirmation in future experiences is needed, some direct implications for clinical practice might be suggested from our findings: (i) early ICD implantation (or at least a wearable ICD), independently of therapeutic optimization, may be considered in arrhythmogenic gene carriers and when LGE ring‐like pattern is present both in DCM and NDLVC patients presenting with LVEF <35%; (ii) among patients starting with LVEF >35%, *TTNtv* becomes the key marker for predicting LVRR. This finding further stresses the need for particular attention to DCM or NDLVC patients presenting with LVEF ≥35%, who should be carefully and closely evaluated with a tailored approach, including polygenic risk calculation in specific families and systematically CMR findings.

Finally, the integration of genetic and CMR imaging data in a multiparametric approach could guide clinicians through a tailored risk assessment and treatment planning, identifying subgroups that require closer clinical evaluation and reassuring those who are likely to experience LVRR.

### Study limitations

This study has some limitations. Although based on patients prospectively enrolled in three registries, it carries the intrinsic limits of retrospective studies, including the lack of central lab for evaluation of echo and CMR images and for reliable LGE quantification. Moreover, patient enrolment was conducted in three tertiary referral centres for cardiomyopathy, introducing a possible selection and referral bias, which may have led to an overrepresentation of certain genetic subtypes, particularly *TTNtv*. Additionally, some arrhythmogenic genes, such as phospholamban, desmosomal genes other than *DSP* and *TMEM43*, were not present in our cohort, partially related to the fact that the genetic panels used in Trieste and Denver did not routinely include these genes during the study period. However, *PLN* and *TMEM43* variants are known to be rare in Italian and North American populations, which makes our cohort representative of the geographical areas involved in the study. Another limitation is that LVRR was defined within 12 months, potentially missing a long‐term decline in LVEF. Furthermore, CMR was performed at a 6‐month interval (with over 70% of patients having CMR within the first 3 months); however, significant changes in LGE within such a short time frame are uncommon in optimally managed DCM patients, and LGE progression has not been associated with adverse LV remodelling. Finally, natriuretic peptide level was not analysed as a predictor of LVRR due to the heterogeneity in measurements (N‐terminal pro‐B‐type natriuretic peptide vs. B‐type natriuretic peptide) and the fluctuations in values during the first 6 months after enrolment, which limits its reliability as a stable predictor.

## Conclusions

In a large, multicentre DCM and NDLVC population well‐characterized through both genetic testing and CMR, the presence of *TTNtv* emerged as an independent predictor of LVRR after 12 months. Conversely, the LGE ring‐like pattern and arrhythmogenic genes were identified as negative predictors of LVRR, particularly in patients starting with LVEF <35%. These results could guide clinicians through a tailored risk assessment and treatment planning.

### Funding

Open access publishing facilitated by University of Trieste, Italy, as part of the Wiley–CRUI‐CARE agreement.

**Conflict of interest**: none declared.

## Supporting information


**Appendix S1.** Supporting Information.
