# Supplementary material for: Integrated role of cardiac magnetic resonance and genetics in predicting left ventricular reverse remodelling in dilated and non‐dilated cardiomyopathy
Source: Eur J Heart Fail. 2025 Apr 29;27(11):2571–81. doi: 10.1002/ejhf.3671 (PMC12765040; doi:10.1002/ejhf.3671)
Supplement: Supplementary file 1 — Appendix S1. Supporting Information. [file EJHF-27-2571-s001.docx]

**Supplementary Material**

**Supplementary Table 1: Characteristics of DCM and NDLVC patients.**

| **Variable** | **DCM (462, 75%)** | **NDLVC (154, 25%)** | ***p*** |
| --- | --- | --- | --- |
| **Clinical characteristics** |  |  |  |
| **Age, years** | 52 ± 13 | 47 ± 16 | **0.0005** |
| **Male sex, n (%)** | 296 (64) | 111 (72) | 0.07 |
| **NYHA class II-IV, n (%)** | 275 (60) | 58 (38) | **<0.0001** |
| **Family history of MVA, n (%)** | 50 (11) | 34 (22) | **0.0007** |
| **Probands, n (%)** | 443 (96) | 143 (93) | 0.2 |
| **Therapy** |  |  |  |
| **Beta-blockers 12-month follow-up, n (%)** | 432 (94) | 123 (80) | **<0.0001** |
| **ACEi 12-month follow-up, n (%)** | 292 (63) | 102 (66) | 0.496 |
| **ARB 12-month follow-up, n (%)** | 62 (13) | 15 (10) | 0.221 |
| **ARNi 12-month follow-up, n (%)** | 68 (15) | 11 (7) | **0.010** |
| **MRA 12-month follow-up, n (%)** | 275 (60) | 62 (40) | **<0.0001** |
| **SGLT2i 12-month follow-up, n (%)** | 24 (5) | 8 (5) | 1.000 |
| **Loop diuretics 12-month follow-up, n (%)** | 152 (33) | 26 (17) | **<0.0001** |
| **Echo characteristics** |  |  |  |
| **LVEF, %** |  |  |  |
| **baseline** | 30±10 | 39±9 | **<0.0001** |
| **12-month follow-up** | 42±11 | 45±9 | **0.002** |
| **LVEDVi, ml/mq** |  |  |  |
| **baseline** | 98±27 | 70±18 | **<0.0001** |
| **12-month follow-up** | 79±26 | 65±16 | **<0.0001** |
| **LAVi, ml/mq** |  |  |  |
| **baseline** | 48±18 | 40±15 | **0.0003** |
| **12-month follow-up** | 38±14 | 39±16 | 0.6 |
| **LVRR, n (%)** | 251 (54) | 63 (41) | **0.004** |
| **Genetic clusters** |  |  | 0.1 |
| **Gene-elusive** | 330 (71) | 99 (64) |  |
| **TTNtv** | 77 (17) | 25 (16) |  |
| **Arrhythmogenic genes** | 29 (6) | 10 (6) |  |
| **Sarcomeric genes** | 16 (3) | 12 (8) |  |
| **Cytoskeletal-Z disk genes** | 7 (2) | 4 (3) |  |
| **Other genes** | 3 (1) | 4 (3) |  |
| **LGE location at CMR** |  |  | 0.4 |
| **No-LGE** | 241 (53) | 84 (55) |  |
| **Any septal** | 109 (24) | 31 (20) |  |
| **Free-wall** | 80 (18) | 25 (16) |  |
| **Ring-like** | 25 (5) | 14 (9) |  |

DCM, Dilated Cardiomyopathy; NDLVC, Non-Dilated Left Ventricular Cardiomyopathy; NYHA, New York Heart Association; ACEi, angiotensin-converting enzyme inhibitors; ARB, angiotensin receptor blockers; ARNi, angiotensin receptor neprilysin inhibitors; MVA, major ventricular arrhythmias; LVEF, left ventricular ejection fraction; LVEDVi, left ventricular end-diastolic volume index; LAVi, left atrial volume index; LVRR, left ventricular reverse remodeling; TTNtv, titin truncating variants; LGE, late gadolinium enhancement.

**Supplementary Table 2: LP/P variant genes in genetic functional clusters**

| **TTNtv** | TTN truncating variants |
| --- | --- |
| **Sarcomeric genes** | MYH7, MYL2, TNNT2, MYPN, ACTC1, TNNC1, TPM1 |
| **Cytoskeleton-Z disk genes** | DES, DMD, NEXN |
| **Arrhythmogenic genes** | FLNC, DSP, LMNA, RBM20 |
| **Other genes** | SCN5A, LAMA4, BAG3, ZNF9 |

**Supplementary Table 3: Predictors of LVRR in univariable analysis.**

| Variable | | *OR* | *95% CI* | *p* |
| --- | --- | --- | --- | --- |
| Genetics | |  |  |  |
| TTNtv – Gene-elusive | | 1.63 | 1.05 - 2.54 | **0.03** |
| TTNtv – Arrhythmogenic genes | 5.61 | | 2.41 - 13.09 | **<0.0001** |
| TTNtv – Sarcomeric genes | | 1.26 | 0.54 -2.95 | 0.6 |
| TTNtv – Cytoskeleton-Z disk genes | | 2.95 | 0.81-10.73 | 0.1 |
| TTNtv – Other genes | | 2.25 | 0.48-10.58 | 0.3 |
| Arrhythmogenic genes – Gene-elusive | | 0.29 | 0.14 - 0.63 | **0.002** |
| Arrhythmogenic genes – Sarcomeric genes | | 0.23 | 0.08 - 0.65 | **0.006** |
| Arrhythmogenic genes – Cytoskeleton-Z disk genes | | 0.53 | 0.13 - 2.21 | 0.4 |
| Arrhythmogenic genes – Other genes | | 0.4 | 0.08 – 2.13 | 0.3 |
| Sarcomeric genes – Gene-elusive | | 1.29 | 0.60 – 2.69 | 0.5 |
| Sarcomeric genes – Cytoskeleton-Z disk genes | | 2.33 | 0.55 – 9.83 | 0.2 |
| Sarcomeric genes – Other genes | | 1.78 | 0.33 – 9.48 | 0.5 |
| Cytoskeleton-Z disk genes – Gene-elusive | | 0.55 | 0.16 – 1.92 | 0.4 |
| Cytoskeleton-Z disk genes – Other genes | | 0.76 | 0.11 – 5.28 | 0.8 |
| Other genes – Gene-elusive | | 0.73 | 0.16 – 3.28 | 0.7 |
| LGE location | |  |  |  |
| Ring-like – no-LGE | | 0.29 | 0.14 – 0.62 | **0.001** |
| Ring-like – Any septal | | 0.37 | 0.17 – 0.81 | **0.02** |
| Ring-like – Free-wall | | 0.31 | 0.13 – 0.68 | **0.004** |
| Any septal – no-LGE | | 0.80 | 0.54 – 1.199 | 0.3 |
| Any septal – Free-wall | | 0.83 | 0.50 – 1.37 | 0.5 |
| Free-wall – no-LGE | | 0.97 | 0.62 – 1.50 | 0.9 |

LVRR, left ventricular reverse remodeling; OR, odds ratio; CI, confidence interval; P, p-value; TTNtv, titin truncating variants; LGE, late gadolinium enhancement.

**Supplementary Table 4: multivariable analysis for LVRR according with LVEF at baseline.**

|  | **Baseline LVEF<35%** | | | | |
| --- | --- | --- | --- | --- | --- |
|  | | *OR* | *95% CI* | *p* |  |
| Ring-like | | **0.24** | **0.09 – 0.65** | **0.005** |  |
| TTNtv* | | 1.09 | 0.59 – 2.00 | 0.8 |  |
| Arrhythmogenic genes* | | **0.24** | **0.07-0.83** | **0.02** |  |
|  | **Baseline LVEF ≥ 35%** | | | | |
|  | | *OR* | *95% CI* | *p* |  |
| Ring-like | | 0.48 | 0.15 – 1.48 | 0.2 |  |
| TTNtv* | | **2.15** | **1.09 – 4.25** | **0.03** |  |
| Arrhythmogenic genes* | | 0.38 | 0.13-1.16 | 0.09 |  |

LVRR, left ventricular reverse remodeling; LVEF, left ventricular ejection fraction; OR, odds ratio; CI, confidence interval; P, p-value; TTNtv, titin truncating variants.

*OR vs other genes.

**Supplementary Figure 1: Flow-chart of study population.**


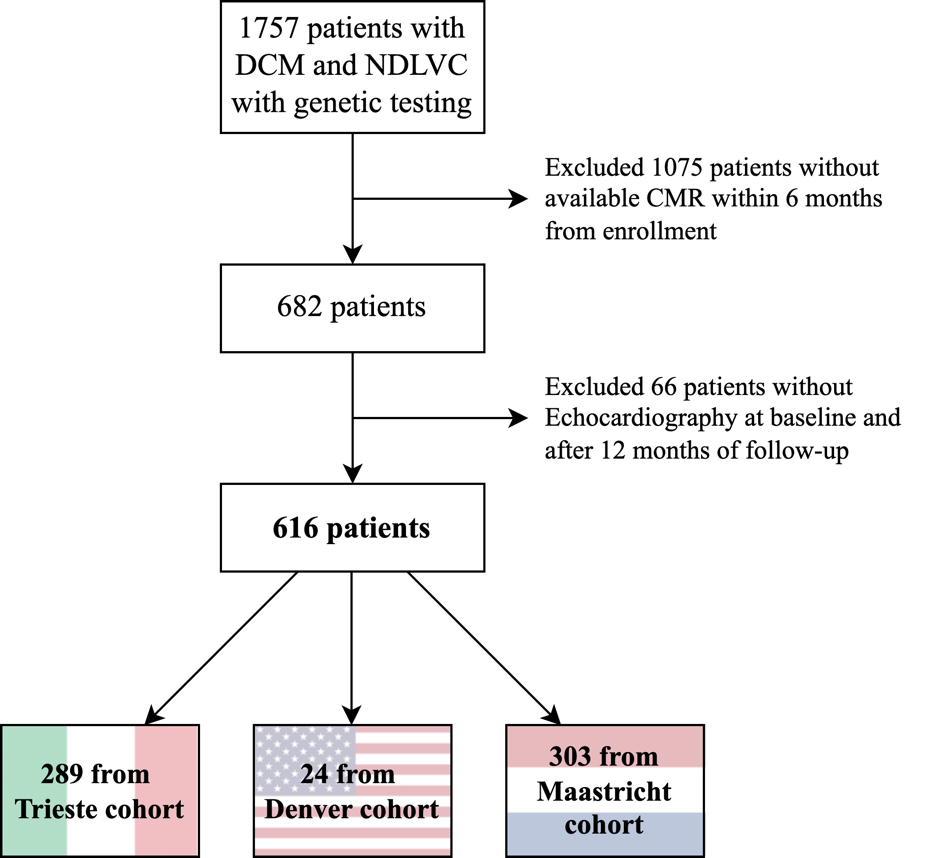


DCM, Dilated Cardiomyopathy; CMR, Cardiac Magnetic Resonance; f-up, follow-up; LGE, late gadolinium enhancement.

**Supplementary Figure 2: Distribution of different gene clusters variants.**

**
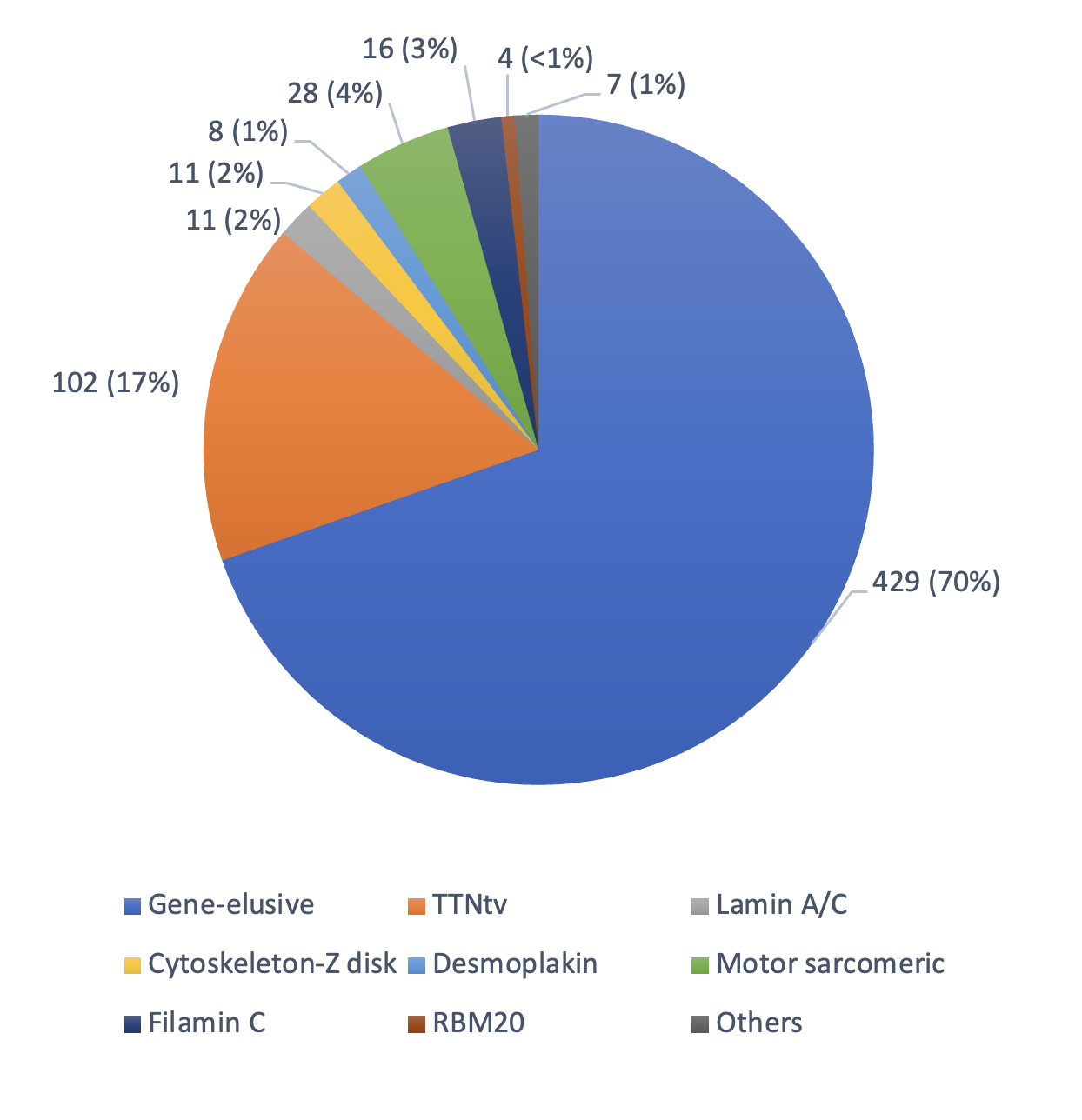
**

TTNtv, titin truncating variants; RBM 20, RNA binding motif protein 20.

**Supplementary Figure 3: Interplay between genetics, LGE distribution and LVRR.**

**
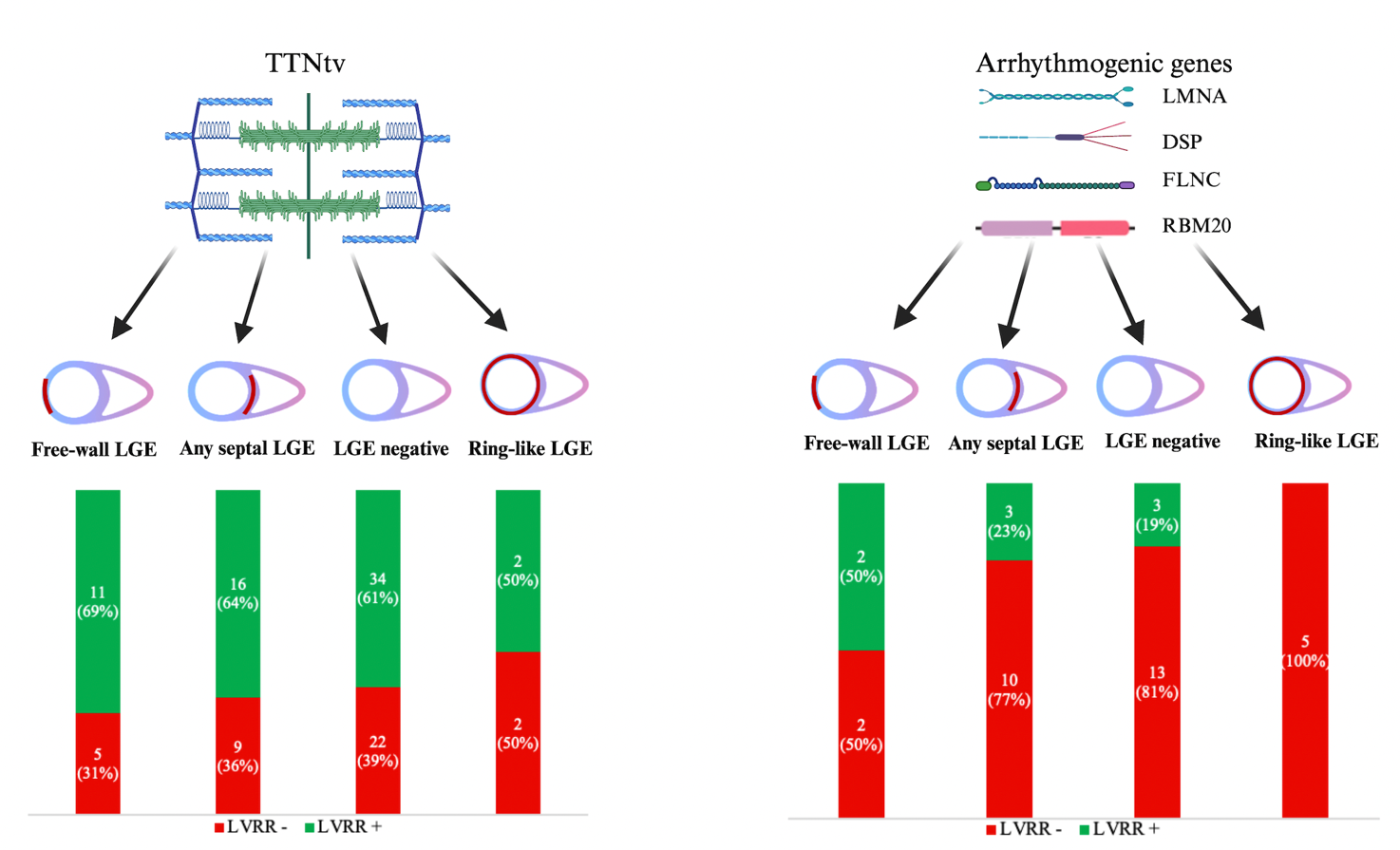
**

LGE, Late Gadolinium Enhancement; LVRR, Left Ventricular Reverse Remodeling; TTNtv, titin truncating variants; LMNA, Lamin A/C, DSP, Desmoplakin; FLNC, Filamin C; RBM 20, RNA binding motif protein 20.

Created in BioRender. https://BioRender.com/f88r886

**Supplementary Figure 4: Cumulative Incidence Curve for D/HTx/LVAD (a) and for SCD/MVA (b) according to LVRR.**

**a)**

**
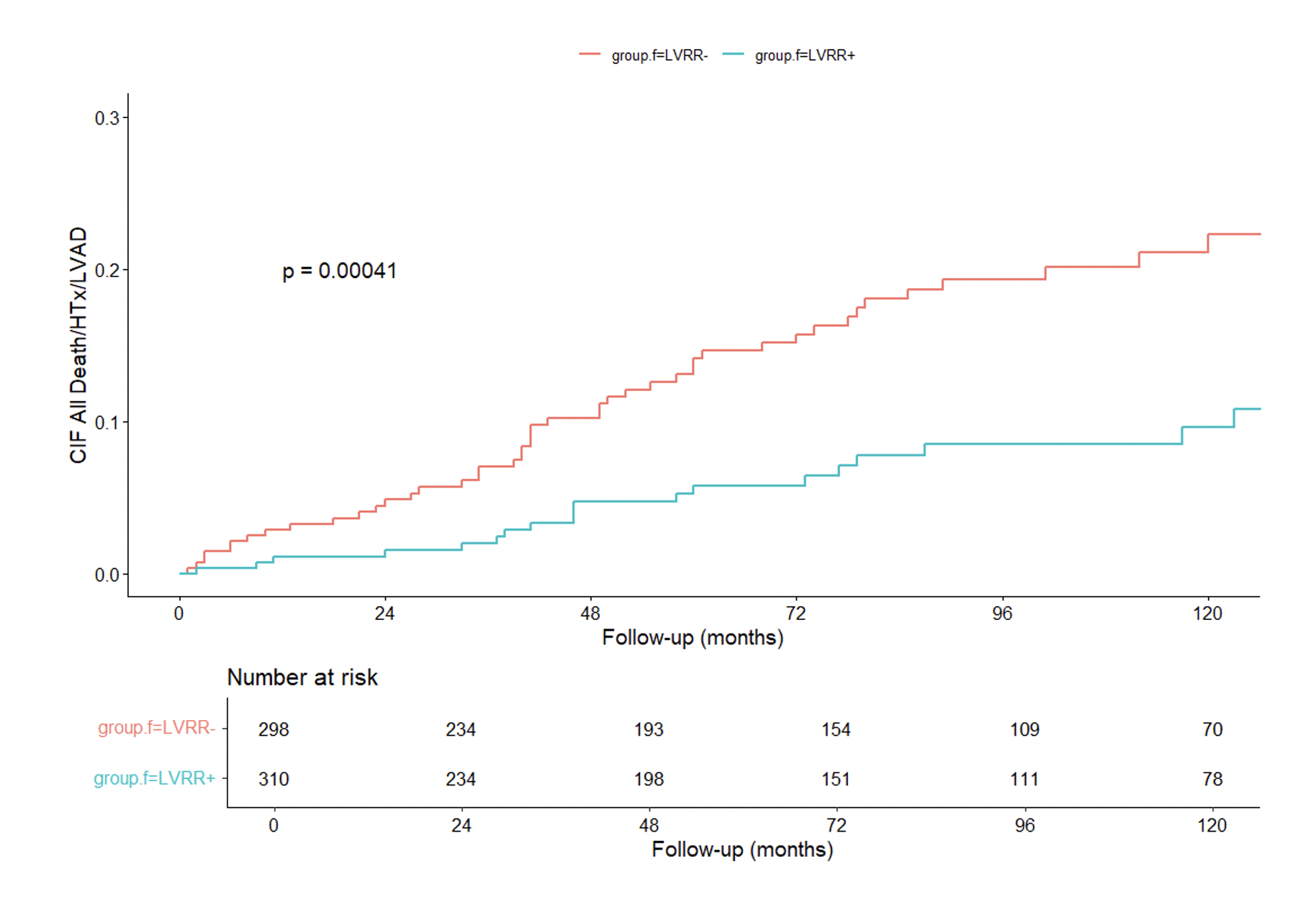
**

**b)**

**
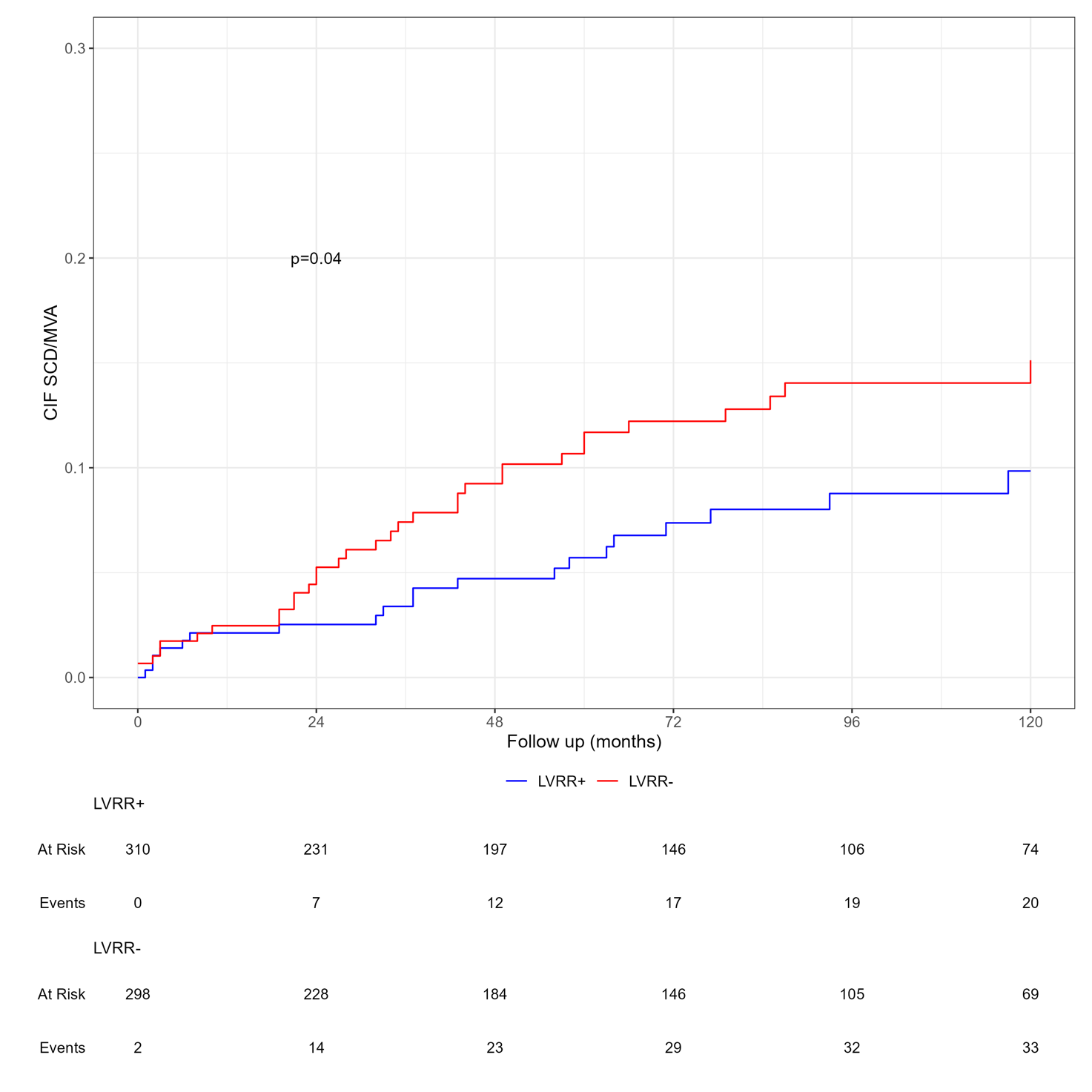
**

D/HT/LVAD, all-cause mortality/heart transplant/left ventricular assist device implantation; SCD/MVA, sudden cardiac death/major ventricular arrhythmias; LVRR, Left Ventricular Reverse Remodeling.

**Supplementary Figure 5: Cumulative Incidence Curve for D/HTx/LVAD (a-b) and for SCD/MVA (c-d) according to LVRR in DCM and NDLVC.**

**
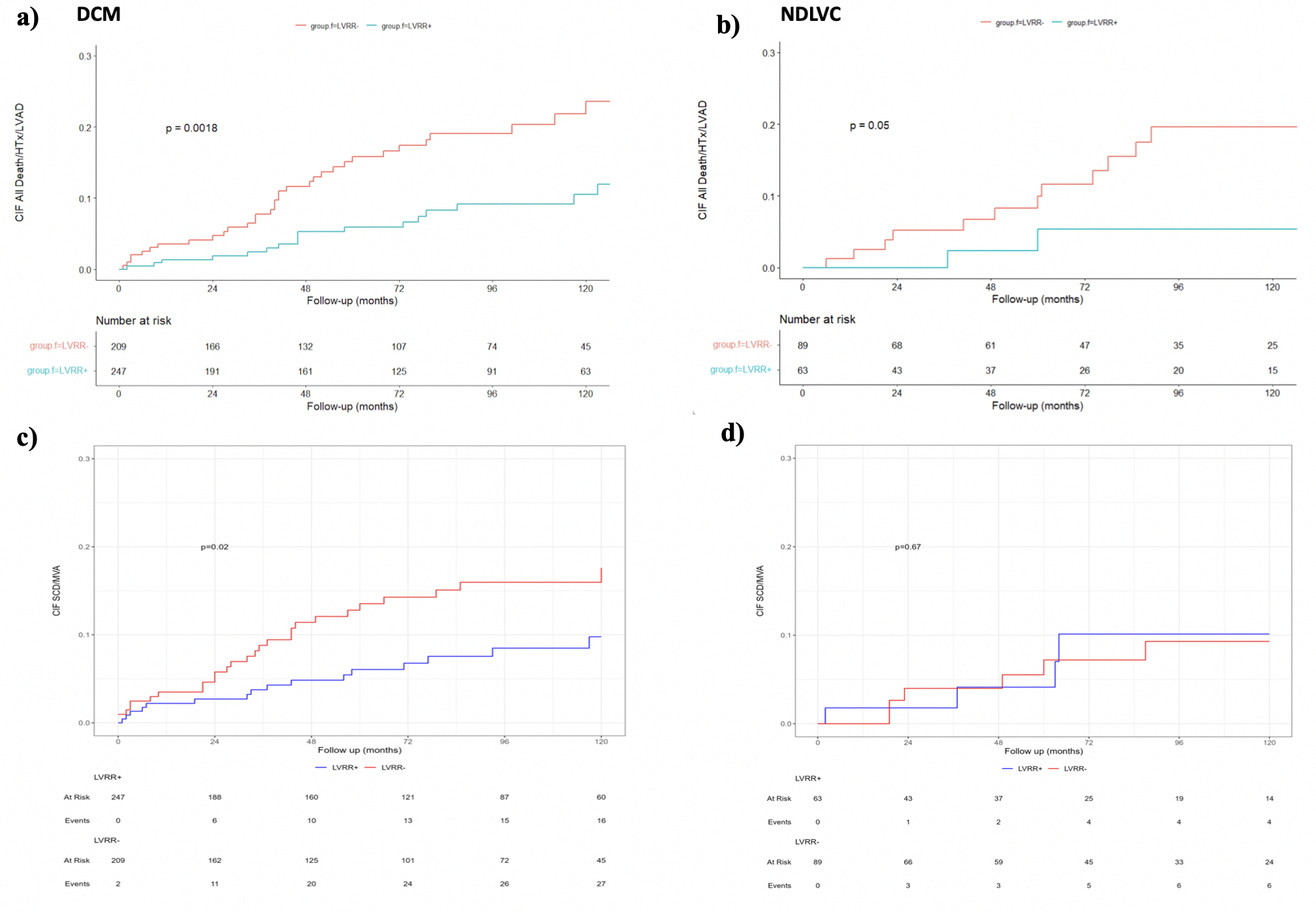
**

D/HT/LVAD, all-cause mortality/heart transplant/left ventricular assist device implantation; SCD/MVA, sudden cardiac death/major ventricular arrhythmias; LVRR, Left Ventricular Reverse Remodeling; DCM, Dilated Cardiomyopathy; NDLVC, Non-Dilated Left Ventricular Cardiomyopathy.
